# A review of air pollution as a driver of cardiovascular disease risk across the diabetes spectrum

**DOI:** 10.3389/fendo.2024.1321323

**Published:** 2024-04-11

**Authors:** Luke J. Bonanni, Sharine Wittkopp, Clarine Long, José O. Aleman, Jonathan D. Newman

**Affiliations:** ^1^ Grossman School of Medicine, New York University (NYU) Langone Health, New York, NY, United States; ^2^ Division of Cardiovascular Disease, Grossman School of Medicine, New York University (NYU) Langone Health, New York, NY, United States; ^3^ Division of Endocrinology, Grossman School of Medicine, New York University (NYU) Langone Health, New York, NY, United States

**Keywords:** air pollution, cardiovascular risk, environmental exposure, inflammation, oxidative stress, particulate matter, prevention

## Abstract

The prevalence of diabetes is estimated to reach almost 630 million cases worldwide by the year 2045; of current and projected cases, over 90% are type 2 diabetes. Air pollution exposure has been implicated in the onset and progression of diabetes. Increased exposure to fine particulate matter air pollution (PM_2.5_) is associated with increases in blood glucose and glycated hemoglobin (HbA1c) across the glycemic spectrum, including normoglycemia, prediabetes, and all forms of diabetes. Air pollution exposure is a driver of cardiovascular disease onset and exacerbation and can increase cardiovascular risk among those with diabetes. In this review, we summarize the literature describing the relationships between air pollution exposure, diabetes and cardiovascular disease, highlighting how airborne pollutants can disrupt glucose homeostasis. We discuss how air pollution and diabetes, via shared mechanisms leading to endothelial dysfunction, drive increased cardiovascular disease risk. We identify portable air cleaners as potentially useful tools to prevent adverse cardiovascular outcomes due to air pollution exposure across the diabetes spectrum, while emphasizing the need for further study in this particular population. Given the enormity of the health and financial impacts of air pollution exposure on patients with diabetes, a greater understanding of the interventions to reduce cardiovascular risk in this population is needed.

## 1 Introduction

Since antiquity, physicians have suspected that air quality could alter human health. Indeed, the Hippocratic Corpus details the importance of clean air, and the philosopher Seneca noted the deleterious health effects of Rome’s contaminated air (1). Research in the past few decades has implicated air pollution in the development of non-communicable diseases, with a strong body of observational and experimental studies establishing a link between air pollution and cardiovascular disease (CVD), encompassing coronary heart disease, heart failure, stroke, peripheral artery disease, and hypertension (2). For example, airborne co-pollutants have been observed to increase hospital admissions for CVD (3, 4). More recently, evidence has implicated air pollution in the onset and progression of type 2 diabetes mellitus (hereafter referred to as diabetes), a widely recognized and significant cardiovascular risk factor (5, 6). Converging lines of evidence in a growing body of literature support the notion that air pollution, especially fine particulate matter (PM_2.5_), can markedly exacerbate CVD risk in patients with diabetes and prediabetes, referred to as the “diabetes spectrum” in this review.

**Figure 1:**
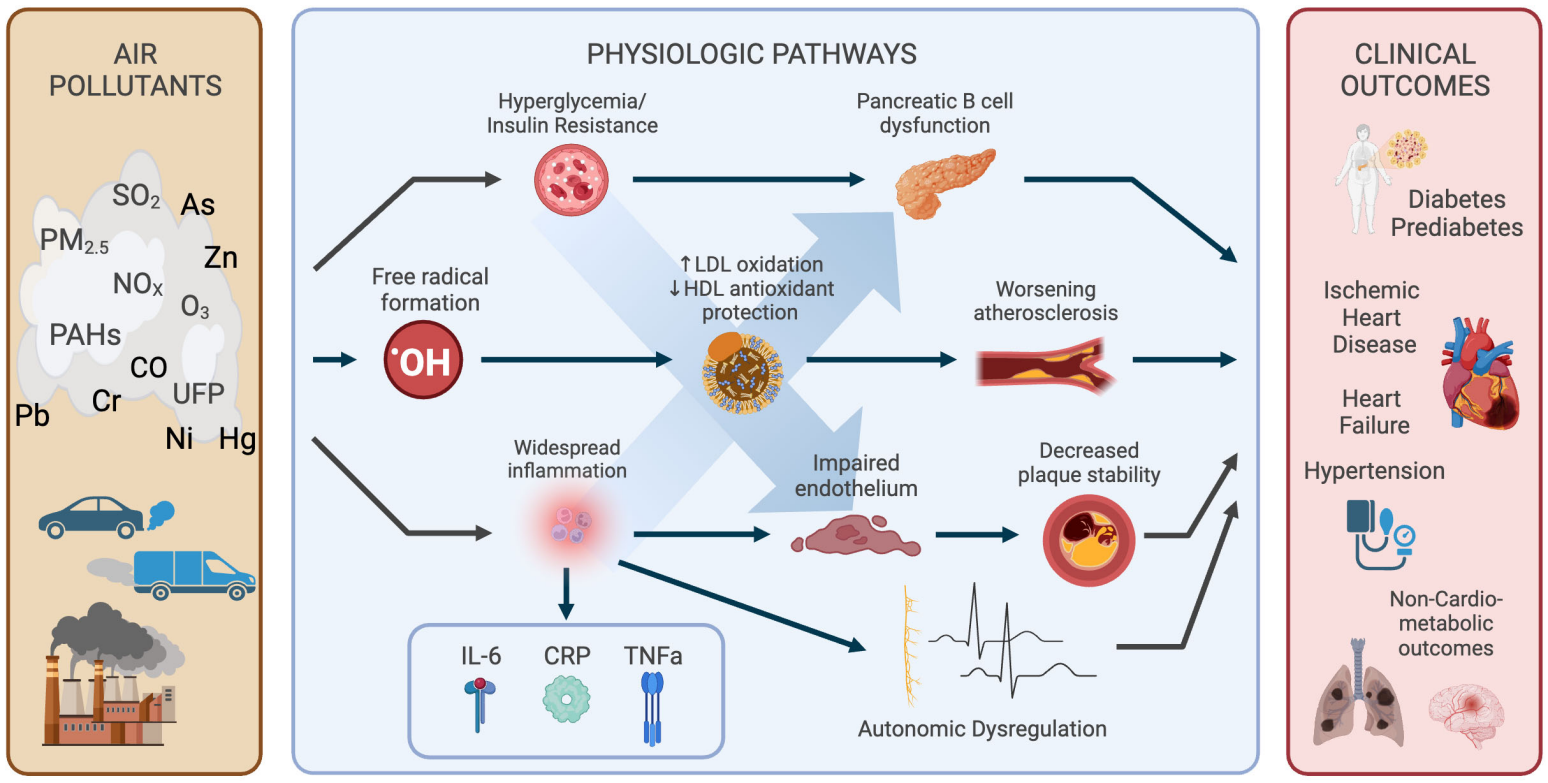
Relationships between air pollution, diabetes and CVD. Multiple physiologic pathways are affected by exposures and drive numerous subclinical and clinical outcomes. Created with BioRender.com.

Globally, exposure to air pollution is the fourth leading risk factor for early death and the fourth leading modifiable risk factor for cardiovascular disease (CVD) (7, 8). In the US, exposure to fine particulate matter (PM_2.5_) has been estimated to result in 8.2 million healthy life-years lost annually from diabetes (9). The magnitude of this ongoing and ubiquitous risk factor for diabetes and CVD would be difficult to overstate. Yet, the problem remains absent from most discussions of risk in health education (10–12). As such, in this review we aim to summarize this existing evidence supporting the relationship between air pollution, diabetes and CVD (**Figure 1**), including the biological mechanisms underlying this phenomenon. Furthermore, this review will discuss potential interventions to reduce air pollution exposure among patients with diabetes and barriers to effective implementation of such interventions. Lastly, this review will identify gaps in the current research landscape and suggest future directions.

## 2 Scope of the problem

### 2.1 Diabetes and CVD

Globally, an estimated 536 million adults have diabetes, either diagnosed or undiagnosed, a number that will increase to 783 million by 2045, driven by expanding populations in middle-income countries (13). In the United States, approximately 37.3 million people have diagnosed or undiagnosed diabetes, with this number expected to increase to over 54.9 million by 2030 (14, 15). Similarly, impaired glucose tolerance has a global prevalence of 464 million that is projected to increase to 638 million by 2045 (16). Whereas in the United States, 96 million adults have prediabetes, estimated to increase to 107 million in 2030 (14, 15). The projected increase in diabetes and prediabetes is partially driven by an aging population in conjunction with climbing obesity rates; increased body mass index (BMI) is strongly related to increased diabetes risk (17). The annual total cost of diagnosed diabetes in the United States is estimated at $327 billion, taking into account healthcare utilization and lost productivity (18). These data make evident the pressing need to identify ways to minimize the incidence and progression of diabetes spectrum disorders, including attention to emerging modifiable risk factors such as environmental exposures.

Like diabetes, CVD is on the rise worldwide, with ischemic heart disease now the second leading cause of morbidity and mortality globally (19). In the United States, nearly 128 million adults live with at least one manifestation of CVD, and 928,741 deaths were attributed to CVD in 2020 alone (20). This heavy burden of CVD in the United States comes at a substantial price, necessitating $407 billion in direct and indirect costs in 2018 (20). By 2060, an estimated 234 million Americans will have CVD, with racial and ethnic minorities bearing the majority of this increased burden (21). As these statistics demonstrate, CVD is and will remain a tremendous problem that will require a multimodal approach to prevention and treatment.

These two chronic conditions separately affect massive populations worldwide with substantial economic and quality of life impact. However, we know there is significant interplay between diabetes and CVD. A long history of prospective cohort studies dating back to the first Framingham Heart Study has established diabetes as a major risk factor for CVD (22). In 2010, the Emerging Risk Factors Collaboration published a meta-analysis of 102 prospective cohort studies, concluding that diabetes confers an approximate 2-fold risk increase for coronary heart disease (95% CI [1.83, 2.19]), with similar risk increases for ischemic (2.27 [1.95, 2.65]) and hemorrhagic stroke (1.84 [1.59, 2.13]) (23). Subsequently, a competing risks analysis using data from 12 Spanish prospective cohorts followed for a median of 10 years found that diabetes increased cumulative risk of cardiovascular death by 1.5-2.5% in both men and women (24). In addition, diabetes appears to not only confer its own risk, but also to accelerate the age-related increase in CVD. A retrospective cohort study found that adults with diabetes develop a high risk of CVD on average 14.6 years sooner versus their counterparts without diabetes (25).

In contrast with older thinking that diabetes increases disease risk only beyond a certain threshold of HbA1c, it appears that CVD risk increases across the continuum of glucose intolerance. An international prospective cohort study of nearly 19,000 adults without diabetes at baseline found that the risk of incident CV events increased by 1.16 [1.11, 1.22] per 1 mmol/L increase in fasting plasma glucose (26). These findings suggest that glucose intolerance should be considered along a continuum, similar to blood pressure (27). There is also evidence linking insulin resistance to CVD. For example, in a prospective cohort study of elderly men in Sweden, insulin resistance was associated with an increased risk of developing congestive heart failure over 7-12 years of follow-up (HR 1.44, 95% CI 1.08-1.93 per 1-SD increase in oral glucose tolerance test glucose level) (28). After 20 years of follow-up in the same cohort, insulin resistance at age 50 was associated with left ventricular dysfunction at age 70 (29). Further supporting a link between diabetes and heart failure, a Swedish cohort study of over 270,000 adults demonstrated that, even with other risk factors in target ranges, patients with diabetes still had an excess risk for hospitalization due to heart failure (HR 1.45 [1.34, 1.57]) (30). Given the significant CVD risk that increases across the diabetes spectrum, developing personal and public strategies to mitigate glucose intolerance at every stage is paramount to preventing excess morbidity and mortality worldwide.

## 3 Air pollution as a risk factor

### 3.1 Background on air pollution

The World Health Organization (WHO) has defined air pollution as “contamination of the indoor or outdoor environment by any chemical, physical, or biological agent that modifies the natural characteristics of the atmosphere” (31). Air pollution is a heterogeneous mixture of particles and gases, much of which is anthropogenic in origin. Nitrogen oxides (NO_x_), including nitrogen dioxide (NO_2_), and carbon monoxide (CO) are generated by fossil fuel combustion, with traffic as a major source. Sulfur dioxide (SO_2_) is generated by fossil fuel combustion for heating homes and generating power (32). Ozone (O_3_) forms in reactions between light and various compounds, including CO and NO_x_. Particulate matter (PM) air pollution is composed of sulfates, nitrogen oxides, ammonia, sodium chloride, black carbon, mineral dust, organic compounds, and products of incomplete combustion of petroleum. PM is generated from many sources, including traffic, industrial, construction, fires, and trash burning, and is typically described in terms of particle size. Coarse PM (PM_10_) is defined as PM with a diameter between 2.5 and 10 μm and fine PM (PM_2.5_) with a diameter less than 2.5 μm; ultrafine PM is PM smaller than 0.1 μm (PM_0.1_). Most studies examine the effects of PM_2.5_, since this is the most widely available data. Thus, the most conclusive effects are seen for this particular pollutant.

### 3.2 Epidemiologic studies on air pollution and diabetes

As early as 1967, researchers probed Public Health Service data to investigate the relationship between air quality and diabetes death rate in urban populations across the US (33). Since then, the body of research on air pollution and diabetes has expanded, especially in the past two decades. This research is summarized in **Table 1**. A 2002 ecological study demonstrated a significant positive correlation between industrial air emissions and diabetes prevalence by state in the US (r = 0.54, p = 5.7x 10^-5^) (34). Another ecological study in 2010 conducted a county-level analysis across the US, showing a 1% increase in county diabetes prevalence per 10 μg/m^3^ per average county increase in PM_2.5_ (β = 0.81 [0.48, 1.07], p < 0.001) (35). Many observational studies over the following decade supported the early ecological findings. A cross-sectional analysis of a Swiss cohort study showed a positive association between 10-year average PM_10_ and NO_2_ exposure and diabetes prevalence, even at levels below the World Health Organization air quality guidelines (OR: 1.40 [1.17 – 1.67], 1.19 [1.03 – 1.38] per 10 μg/m^3^ increase in pollutant, respectively) (45). A cross-sectional study of 69,000 adults in China without a prior history of diabetes demonstrated that for each standard deviation increase in 3-year average concentration of PM_2.5_ there were increased odds of diagnosed diabetes (OR: 1.04 [1.01, 1.07]) by fasting blood (46). Another cross-sectional study of 11,847 adults in China found that annual average PM_2.5_ exposure was associated with diabetes prevalence (PR: 1.14 [1.08, 1.20] for a 41.1 μg/m^3^ increase in PM_2.5_), with a greater effect seen in subjects who were male, smoking, elderly, or had high BMI or less education (38).

**Table 1:** Studies of links between air pollution and DM incidence and prevalence.

First Author (Year)	Design	Location	Population/Health Data Source	Pollutants	Relevant Outcomes
Lockwood (2002) (34)	Ecological; state-level	United States	Behavioral Risk Factor Surveillance System, 184,450 respondents	State industrial air pollutant releases	DM prevalence; linear regression r = 0.54, p < 0.0001
Pearson et al. (2010) (35)	Ecological; county-level	United States	CDC DM statistics, nationwide	Annual PM_2.5_	1% increase in DM prevalence for 10 μg/m^3^ increase in PM_2.5_, β = 0.81, p < 0.001
Eze et al. (2014) (36)	Cross-sectional	Switzerland	SAPALDIA cohort, 6,392 adults	10-year PM_10,_ NO_2_	DM prevalence per 10 μg/m^3^ increase in: PM_10_ OR 1.40, NO_2_ 1.19
Li et al. (2023) (37)	Cross-sectional	Southwest China	CMEC cohort, 69,210 adults	3-year PM_2.5_, BC, NH_4_^+^, NO_3_^-^, OM, soil	DM prevalence at follow-up per SD increase in: PM_2.5_ OR 1.08, BC 1.07, NO_3_^-^ 1.08, OM 1.09, soil 1.09; Nonsignificant positive association for NH_4_^+^
Liu et al. (2016) (38)	Cross-sectional	China	CHARLS cohort, 11,847 adults	Annual PM_2.5_	DM prevalence per IQR increase in PM_2.5_ PR 1.14
Qiu et al. (2018) (39)	Prospective cohort	Hong Kong	EHS cohort, 61,447 older adults	Annual PM_2.5_ for 10 years	DM prevalence per IQR increase in PM_2.5_ OR 1.06 DM incidence per IQR increase in PM_2.5_ HR 1.15
Chen et al. (2013) (14)	Prospective cohort	Ontario	National Population Health Survey and Canadian Community Health Survey, 62,012 adults, no baseline DM	6-year PM_2.5_	DM incidence per 10 μg/m^3^ increase in PM_2.5_ HR 1.11
Hansen et al. (2016) (40)	Prospective cohort	Denmark	Danish Nurse Cohort, 24,174 female nurses, no baseline DM	Annual PM_2.5_, PM_10_, NO_2_, NO_x_ for 15 years	DM incidence per IQR increase in: PM_2.5_ HR 1.11. Nonsignificant positive associations for NO_2_, NO_x_
Krämer et al. (2010) (41)	Prospective cohort	Germany	SALIA cohort, 1,775 adult women, no baseline DM	Annual PM_10_, NO_2_, Traffic-related PM for 16 years	DM incidence per IQR increase in: traffic-related PM HR 1.15, NO_2_ HR 1.34. Nonsignificant positive association for PM_10_
Puett et al. (2011) (42)	Prospective cohort	United States	NHS and HPFS cohorts, 89,460 adults, no baseline DM	Annual PM_10_, PM_2.5_, PM_10-2.5_	DM incidence per IQR increase in pollutant. Nonsignificant positive associations for all pollutants.
Eze et al. (2014) (36)	Prospective cohort	Switzerland	SAPALDIA cohort, 2,631 adults, no baseline DM	Annual NO_2_ at beginning and end of 9-years	DM incidence per IQR increase in NO_2_, nonsignificant negative association
Coogan et al. (2016) (43)	Prospective cohort	United States	BWHS cohort, 43,003 black women, no baseline DM	Annual NO_2_ for 10 years	DM incidence per IQR increase in NO_2_, nonsignificant negative association
Andersen et al. (2012) (44)	Prospective cohort	Denmark	Danish DCR cohort, 51,818 middle-aged adults, no baseline DM	35-year NO_2_	DM incidence per IQR increase in NO_2_, nonsignificant positive association

Outcomes column reports results for fully adjusted models when applicable. Means reported for significant results.

The findings from these cross-sectional studies have been recapitulated in prospective data with mixed results. Particulate matter-associated diabetes incidence was investigated in a prospective cohort study of over 61,000 elderly Hong Kong residents without diabetes at baseline followed from 1998 to 2010. The analysis showed an increased risk of incident diabetes (HR: 1.15 [1.05, 1.25]) per 3.2 μg/m^3^ increase in average annual PM_2.5_ exposure (39). A Canadian cohort study followed 62,000 adults without diabetes in Ontario for up to 15 years, during which time a 10 μg/m^3^ increase in average PM_2.5_ exposure was associated with an increased risk of incident diabetes (HR 1.11 [1.02, 1.21]) (47). A Danish prospective cohort study from 1993 until 2013 found that annual average PM_2.5_ was significantly associated with increased diabetes incidence (HR: 1.11 [1.02, 1.22]), especially in patients with obesity (40). Additionally, a 16-year-long cohort study of women in Germany without diabetes at baseline demonstrated a 15% [4%, 27%] increase in the risk of incident diabetes per 1 interquartile range increase in traffic-related PM (a composite of particles derived from traffic) and NO_2_ exposure, but no significant risk increase due to PM_10_ exposure (41). In contrast, an analysis of participants in the Nurses’ Health Study and the Health Professionals Follow-Up Study found no significant association between 1-year average PM_2.5_ or PM_10_ and incident diabetes, although the direction of effect was weakly positive (1.03 [0.96, 1.10] and 1.04 [0.99, 1.09] for PM_2.5_ and PM_10_, respectively) (42). Differences across these studies may be due to varying exposure assessments or particle compositions, or due to differences in cohort characteristics (e.g., diet, BMI). Thus, despite conflicting results, the majority of studies support an association between particulate matter air pollution exposure and diabetes incidence.

Similar to particulate matter, there are differing reports on associations between NO_2_ exposure and diabetes. NO_2_ exposure was studied in a prospective study of 2,631 Swiss adults without baseline diabetes followed from 2002 to 2011; results showed no significant association between average annual NO_2_ exposure and diabetes incidence (RR: 0.87 [0.60, 1.22]). However, few incident diabetes cases in the cohort may have diminished the ability to detect an effect (36). A similar negative finding for NO_2_ was found in a prospective cohort of African American women residing in US cities (HR: 0.90 [0.82 – 1.00] for a 9.7 ppb increase in NO_2_ in the fully adjusted model); this negative result was purported to be due to confounding by socioeconomic status (SES) given the inverse correlation between neighborhood SES and NO_2_ in this cohort (43). Among women in the Nurses’ Health Study, there was, however, a significant association between increased proximity to a roadway and developing diabetes (HR: 1.14 [1.03, 1.27] for living < 50 m vs. ≥00 m from a roadway). Because motor vehicles are a major generator of NO_2_, and proximity to roadways has been used as a surrogate marker for exposure, the authors suggest this result may support a link between NO_2_ and diabetes in females (42). Similar to the Nurses’ Health Study results, a Danish prospective cohort study using a national public register found a nonsignificant but positive association between NO_2_ exposure and confirmed diabetes (HR: 1.04 [1.00, 1.08] per 4.9 μg/m^3^ increase in average NO_2_), with the strongest associations in women and subjects with elevated waist-to-hip ratio (44). A cross-sectional study of respiratory clinic patients in two Canadian cities found a positive association between NO_2_ exposure and diabetes diagnosis but only for women (OR: 1.04 [1.00, 1.08]) (48). A comparable cross-sectional study in the Netherlands showed a non-significant association between increasing levels of NO_2_ exposure and diabetes diagnosis, with the direction of effect stronger in women (OR: 1.48 [1.07, 2.04]) (49). Given that air pollution is a complex mixture of particles and gases, it is challenging to interpret studies of the health effects of individual component pollutants. Regardless, evidence continues to mount in support of the association between increasing air pollution exposure and increases in incident and prevalent diabetes.

#### 3.2.1 Short-term effects of air pollution on diabetes and glucose homeostasis

In addition to the literature supporting associations between air pollution exposure and incidence of diabetes, there is growing literature showing worsening of glucose metabolism with air pollution exposure for patients that already have a diabetes diagnosis. Furthermore, short-term exposures to air pollution, over days to weeks, seem to cause dysregulated glucose homeostasis, even among those without diabetes. The following studies examining short-term air pollution exposures and diabetes are summarized in **Table 2**.

**Table 2:** Studies of short-term effects of air pollution on glucose homeostasis.

First Author (Year)	Design	Location	Population/Health Data Source	Pollutants	Relevant Outcomes
Li et al. (2018) (50)	Cross-sectional	China	Xinqiao Hospital, 2,840 hospitalized DM patients	15-day SO_2_, CO, NO_2_	LOS per 10 μg/m^3^ rise in: SO_2_ 0.487 days increase, CO 0.013 days increase, NO_2_ 0.359 days decrease
Yitshak-Sade et al. (2015) (51)	Retrospective cohort	Israel	Clalit Health Services, 131,882 adult members	24–72-hour SO_2_, NO_2_	FBG increase: per IQR increase in: NO_2_ in healthy adults 0.40%, prediabetes 0.56%, diabetes 1.08%; SO_2_ in healthy adults 0.29%, prediabetes 0.20%, diabetes 0.33%
Yitshak-Sade et al. (2015) (51)	Retrospective cohort	Israel	Clalit Health Services, 73,117 adult members	1-7-day and 12-week PM_10_, PM_2.5_	Overall FBG increase per IQR increase in: 12-week PM_10_ 0.30%. Nonsignificant positive association for 12-week PM_2.5_ and negligible associations for 1-7-day PM_10_ or PM_2.5_. In people with diabetes, HbA1c increase per IQR increase in: 12-week PM_10_ 3.58%, PM_2.5_ 2.93%
Lucht et al. (2018) (52)	Prospective cohort	Germany	HNR study cohort, 7,108 adults, no baseline DM	28-day PN_AM_, PM_2.5_	FBG increase per IQR increase in: PN_AM_ 0.64 mg/dL, PM_2.5_ 0.91 mg/dL
Li et al. (2018) (50)	Prospective cohort	United States	Framingham cohorts, 5,958 adults, no baseline DM	1-3-7-day BC, NO_x_, PM_2.5_, O_3_, SO_4_^2-^	FBG increase: 7-day BC approx. 0.5%, 7-day NO_x_ approx. 0.5%. Other results nonsignificant or neglibible
Chen et al. (2016) (53)	Prospective cohort	China	Kailuan cohort, 27,685 adults	4-day NO_2_, SO_2,_ PM_10_	FBG increase per 100 μg/m^3^ increase in: NO_2_ 0.53 mmol/L, SO_2_ 0.17 mmol/L, PM_10_ 0.11 mmol/L
Brook et al. (2013) (54)	Experimental	United States	25 adults, no DM	5 days PM_2.5_ from ambient urban air, 4-5-hr/d	HOMA-IR increase per 10 μg/m^3^ increase in PM_2.5_ 0.7
Chen et al. (2016) (53)	Cross-sectional	United States	BetaGene cohort, 1,023 adult Mexican Americans, personal or family history of GDM	37-40-days PM_2.5_, NO_2_	HOMA-IR increase for PM_2.5_ β = 6.99 p = 0.002, NO_2_ β = 6.63, p = 0.009
Li et al. (2018) (50)	Randomized, double-blind, crossover trial	China	55 college students, no DM	9-day PM_2.5_ during trial	HOMA-IR increase per 10 μg/m^3^ increase in PM_2.5_ approx. 10%

Outcomes column reports results for fully adjusted models when applicable. Means reported for significant results.

A cross-sectional study of 2,840 patients with diabetes hospitalized from 2013-2016 in Chongqing, China, investigated the impact of short-term, 15-day average air pollution exposure on length of stay and cost of admission. The study authors found a positive correlation between a 10 μg/m^3^ increase in sulfur dioxide (SO_2_) and carbon monoxide (CO) exposure and prolonged length of stay, increased by 0.487 days [0.318, 0.656] and 0.013 days [0.003, 0.022], respectively, with a concordant increase in the cost of hospitalization (50). In Israel, a retrospective study between 2001-2012 of over 1 million fasting blood glucose tests from approximately 130,000 patients found a significant positive association between fasting blood glucose and 24-72 hour averages for NO_2_ and SO_2_ in all patients regardless of diabetes status. A 6.36 ppb increase in NO_2_ was associated with a 0.40% [0.31%, 0.50%] increase in fasting glucose in patients without diabetes, 0.56% [0.40%, 0.71%] in those with prediabetes, and 1.08% [0.86%, 1.29%] in those with diabetes.; for a 1.17 ppb increase in SO_2_ fasting glucose increased by 0.29% [0.22%, 0.36%], 0.20% [0.10%, 0.31%], 0.33% [0.14%, 0.52%], in these same groups (51). A similar retrospective study in the same Israeli population found that 12-week average PM_10_ and PM_2.5_ exposure was associated with increased fasting blood glucose in all patients (0.30% [0.153%, 0.452%]; 0.02% [-0.12%, 0.18%], respectively) and this increase was more pronounced in those with diabetes (0.57% increase [0.29%, 0.85%], 0.41% increase [0.12%, 0.69%]). Also, HbA1c increases were found in patients with diabetes (3.58% [1.03%, 6.20%]; 2.93% [0.35%, 5.59%] for PM_10_ and PM_2.5_ respectively). The 1-7 day average PM_10_ and PM_2.5_ exposure windows had no or negligible association with fasting blood glucose and HbA1c (55).

Prospective data have corroborated the retrospective data suggesting short-term effects of air pollution on blood glucose. A German prospective cohort study between 2000-2008 of 7,108 adults without diabetes at baseline evaluated short-term associations between air pollution exposure and fasting blood glucose levels and HbA1c. Increases in 28-day average accumulation mode particle number (PN_AM_, PM between 0.1-1μm in aerodynamic diameter) and PM_2.5_ concentrations were both positively associated with increasing blood glucose (0.64 mg/dL [0.07, 1.21] per 2,142.3 n/mL increase and 0.91 mg/dL [0.38, 1.44] per 5.7 μg/m^3^ increase, respectively) (52). In the US Framingham Heart Study, increased 7-day moving average BC and NO_x_ exposures were positively associated with higher fasting glucose among adults without diabetes. In contrast, an increased short-term O_3_ exposure was inversely associated with blood glucose (exact numbers not provided by the study authors) (56). A prospective cohort study of approximately 28,000 adults in China followed from 2006-2008 found that a 100 μg/m^3^ increase in the 4-day average of NO_2_, SO_2_, or PM_10_ exposure was associated with elevated fasting blood glucose (0.53 mmol/L [0.42, 0.65], 0.17 mmol/L [0.15, 0.19], 0.11 mmol/L [0.07, 0.15], respectively), with increased elevations among female, elderly, or overweight subjects (53). A recent study of 2 large Indian cities (Chennai and Delhi) found that a 10 μg/m^3^ difference in 1-month average exposure to PM_2.5_ was associated with a 0.40 mg/dL increase in fasting plasma glucose (95% CI 0.22 to 0.58) and 0.021 unit increase in HbA1c (95% CI 0.009 to 0.032) (57).

Some studies have been conducted on short-term air pollution exposure and glucose metabolism using the homeostasis model assessment of insulin resistance (HOMA-IR). In one study, 25 adults without diabetes who resided in rural Michigan were exposed to urban ambient air for 4-5 hours per day for 5 days. HOMA-IR was measured before, during, and after the air pollution intervention. A positive correlation was found between each 10 μg/m^3^ increase in measured PM_2.5_ exposure and study subjects’ HOMA-IR (+0.7 [0.1, 1.3]). A 3.5 μg/m^3^ increase in PM_2.5_ was associated with worsening HOMA-IR (+0.25 [0.04, 0.46], indicating potential adverse effects even at low concentrations of PM_2.5_ (54). A cross-sectional analysis of Mexican American women with a personal or family history of gestational diabetes but without diabetes at the time of the study revealed that up to 40 days of daily PM_2.5_ exposure and up to 37 days of daily NO_2_ exposure were associated with increased HOMA-IR (β = 6.99, p = 0.002 for PM_2.5_; β= 6.63, p = 0.009 for NO_2_). However, no significant associations were found for O_3_ exposure (58). Last, a clinical trial testing 48 hours of portable air cleaner (PAC) intervention in healthy college students in China showed an approximately 10% increase in HOMA-IR per 10 μg/m3 increase in PM_2.5_ (exact numbers not provided) (59).

In summary, the collective evidence supports short-term associations between air pollution exposure and fasting glucose and dysregulated glucose metabolism evidenced by HOMA-IR, but not HbA1c.

#### 3.2.2 Long-term effects of air pollution on diabetes and glucose metabolism

Long-term exposures to air pollution also have been shown to affect glucose homeostasis. Studies examining this phenomenon have been summarized in **Table 3**. Cross-sectional analyses of a Chinese cohort found 1-year average PM_2.5_ to be positively associated with both elevated fasting glucose (0.26 mmol/L increase [0.19, 0.32]) and HbA1c (0.08% increase [0.06%, 0.10%]) for a large, 41.1 μg/m^3^ increase in PM_2.5_ (38). Furthermore, a secondary analysis of a Taiwanese cohort found 1-year average PM_2.5_, PM_10_, O_3_, and NO_2_ to be positively associated with fasting glucose and HbA1c; a 20.42 μg/m^3^ increase in PM_2.5_ was associated with 34.6 mg/dL [16.5, 52.7] increase in fasting glucose and 2.1% [1.5, 2.7] increase in HbA1c (60). The air pollution exposures experienced by this cohort were substantially above the WHO guidelines. A subsequent cross-sectional study of 2,895 adults in the Dunkirk and Lille areas of France, regions with relatively low concentrations of air pollution, found that HbA1c was 0.044% higher [0.021%, 0.067%] with a 2 μg/m^3^ increase in annual mean PM_10_ and 0.031% higher [0.010%, 0.053%] with a 5 μg/m^3^ increase in annual mean NO_2_. However, neither NO_2_ nor PM_10_ were significantly associated with diabetes prevalence, likely due to a low number of patients with diabetes in the study sample. Moreover, neither pollutant had an association with fasting blood glucose (61).

**Table 3:** Studies of long-term effects of air pollution on glucose homeostasis.

First Author (Year)	Design	Location	Population/Health Data Source	Pollutants	Relevant Outcomes
Liu et al. (2016) (38)	Cross-sectional	China	CHARLS cohort, 11,847 adults	Annual PM_2.5_	FBG increase per 41.1 μg/m^3^ increase in PM_2.5_ 0.26 mmol/L. HbA1c increase per 41.1 μg/m^3^ increase in PM_2.5_ 0.08%
Chuang et al. (2011) (60)	Cross-sectional	Taiwan	SEBAS sample, 1,023 older adults	Annual PM_10_, PM_2.5_, O_3_, NO_2_, SO_2_	FBG increase per IQR increase in: PM_10_ 22.88 mg/dL, PM_2.5_ 36.55 mg/dL, O_3_ 21.10 mg/dL, NO_2_ 17.03 mg/dL, SO_2_ positive but nonsignificant. HbA1c increase per IQR increase in: PM_10_ 1.40%, PM_2.5_ 2.24%, NO_2_, O_3_, and SO_2_ positive but nonsignificant
Riant et al. (2018) (61)	Cross-sectional	France	ELISABET survey, 2,895 adults	Annual PM_10_, NO_2_	HbA1c increase per 2 μg/m^3^ increase in PM_10_ 0.044%, per 5 μg/m^3^ increase in NO_2_ 0.031%. Nonsignificant associations for FBG
Lucht et al. (2018) (52)	Prospective cohort	Germany	HNR study cohort, 7,108 adults, no baseline DM	91-day PN_AM_, PM_2.5_, PM_10_, NO_2_	FBG increase per IQR increase in: PN_AM_ 0.67 mg/dL, PM_2.5_ 0.81 mg/dL. HbA1c increase per IQR increase in: PN_AM_ 0.09%, PM_2.5_ 0.07%, PM_10_ 0.04%. No association for NO_2_
Brook et al. (2013) (62)	Prospective cohort	Canada	Canadian census mortality follow-up study, 2.1 million adults	5-year PM_2.5_	Diabetes related mortality per 10 μg/m^3^ increase in PM_2.5_ HR 1.49
Hwang et al. (2022) (63)	Cross-sectional	South Korea	Seoul National University Health Examination Center, 4,251 adults	Annual PM_10_ exposure	HOMA-IR increase per 11 μg/m^3^ increase in PM_10_ 14%
Honda et al. (2017) (64)	Prospective cohort	United States	NSHAP cohort, 4,121 older adults	2-year PM_2.5_ NO_2_	In people with diabetes, HbA1c increase per IQR increase in: PM_2.5_ 1.8%, NO_2_ 2.0%
Khafaie et al. (2018) (65)	Cross-sectional	India	WellGen study cohort, 1,213 young adults with DM	1-year PM_10_	HOMA-IR increase per 43.83 μg/m^3^ increase in PM_2.5_ 4.89%
Chen et al. (2016) (53)	Cross-sectional	United States	BetaGene cohort, 1,023 adult Mexican Americans, personal or family history of GDM	Annual PM_2.5_, NO_2_, O_3_	HOMA-IR increase for PM_2.5_ β = 5.81 p = 0.016, no association for NO_2_ or O_3_
Wolf et al. (2016) (66)	Cross-sectional	Germany	KORA study, 2,944 adults	2-year PM_2.5_, NO_2_	HOMA-IR increase per IQR increase in: PM_2.5_ 15.6%, NO_2_ 19.2%. Insulin increase per IQR increase in: PM_2.5_ 14.5%, NO_2_ 17.2%

Outcomes column reports results for fully adjusted models when applicable. Means reported for significant results.

Large prospective cohort studies repeatedly demonstrate the long-term effects of air pollution exposure on diabetes outcomes. A German prospective cohort study without baseline diabetes demonstrated that 91-day average exposure to PN_AM_ and PM_2.5_ was associated with increased random blood glucose and, more strongly, with increased HbA1c. There was a 0.67 mg/dL [0.10, 1.24] and 0.81 mg/dL [0.05, 1.58] increase in random blood glucose (adjusted for time since last meal) per interquartile range (IQR) increase in PN_AM_ (1,352.7 n/mL) and PM_2.5_ (4.0 μg/m^3^), respectively. In this German study, HbA1c increased by 0.09% [0.07, 0.11] and 0.07% [0.04, 0.10] per IQR increase of each pollutant, respectively) (52). A census data analysis of 2.1 million randomly selected Canadian adults, followed from 1991 to 2001, found that a 10 μg/m^3^ increase in average PM_2.5_ exposure over a 5-year period was associated with a hazard ratio of 1.49 [1.37, 1.62] for diabetes-related mortality. This association was consistent across subgroups of age, sex, education, income, community, and at low concentrations of PM_2.5_ (<5 μg/m^3^). The risk of diabetes-related mortality was most pronounced in participants with lower SES as well as aboriginal ancestry (62). Similar effects on HbA1c have been observed in U.S. cohort studies. For example, in a probability sample of U.S. adults with diabetes over 57 years of age (n= 4121) followed from 2005 to 2011, a 3.7 μg/m^3^ increase in 2-year moving average PM_2.5_ was associated with an increase in HbA1c of 1.8% ± .6% (p<0.01). In subjects without diabetes, a significant positive association with HbA1c was found for NO_2_ exposure (0.8% ± .2%, p<0.01) (64). The prospective cohort study from Chennai and Delhi showed that a 1-year increase in PM_2.5_ exposure of 10 μg/m^3^ was associated with increased HR for incident diabetes (1.22 [1.09, 1.36]), with similar significant estimates for 1.5-year and 2-year exposures as well (57).

In addition to the long-term associations with HbA1c, long-term exposures have been associated with measures of insulin resistance. A cross-sectional study of long-term air pollution exposure in Korea found that the relationship between HOMA-IR and PM_10_ observed in studies of short-term air pollution exposures retained significance even with rigorous adjustment for visceral adiposity, with a dose-dependent increase in HOMA-IR by 14% [8%, 21%] for men and 14% [7%, 21%] for women, per 11 μg/m^3^ increase in PM_10_ (63). Other cross-sectional studies have found similar associations with HOMA-IR. A cross-sectional study investigating the effect of PM_10_ exposure in young-onset (before age 46) patients with diabetes at a clinic in India found that a 43.83 μg/m^3^ increase in 1-year average PM_10_ exposure was associated with increased HOMA-IR of 4.89% [0.59%, 9.37%], with a significantly greater effect in female and patients with obesity (65). In the cross-sectional analysis of Mexican American women discussed previously, annual PM_2.5_ exposure was associated with increased HOMA-IR (beta coefficient 5.81, p = 0.016), without significant associations for annual NO_2_ or O_3_ exposures (58). A German prospective cohort study of nearly 3,000 adults with and without diabetes/prediabetes found that a 7.9 μg/m^3^ increase in 2-year average PM_2.5_ exposure was associated with increased HOMA-IR (15.6% [4.0%, 28.6%]) and insulin (14.5% [3.6%, 26.5%]). In contrast, an 11.9 μg/m^3^ increase in 2-year average NO_2_ exposure was associated with increases in HOMA-IR by 19.2% [7.7%, 31.6%], insulin by 17.2% [6.6%, 29.0%], glucose by 1.7% [0.1%, 3.3%], and leptin by 15.3% [6.8%, 24.5%]. However, there was no association between either pollutant and HbA1c (66).

While effect sizes have varied across cohorts and pollutants, the directionality of the relationships between long-term air pollution exposures and HbA1c remains generally consistent. If short-term air pollution exposure induces hyperglycemia, then we would expect increases in medium- and long-term exposure to have the effect of raising HbA1c. As expected, most studies to date support that months-long exposures are more strongly associated with HbA1c, indicating a potential cumulative effect of shorter-term air pollution exposure.

### 3.3 Diabetes may confer increased vulnerability to the cardiovascular effects of air pollution

Patients with diabetes appear to be more vulnerable to the vasculotoxic effects of air pollution exposure. Studies examining this potential predisposition have been summarized in **Table 4**. A cross-sectional analysis using Illinois Medicare data from 1988-1994 found that a 10 μg/m^3^ increase in ambient PM_10_ exposure in the 24 hours prior to admission was associated with a 2.01% [1.40%, 2.62%] increase in hospital admission for CVD in patients with diabetes, a two-fold higher increase in CVD hospitalizations than that observed for adults without diabetes (67). A case-crossover study examining emergency department visits in Atlanta reported increased odds of visits for dysrhythmia with increasing NO_2_ exposure for people with diabetes (OR 1.158 [1.046, 1.282]) compared to people without diabetes (1.014 [0.988, 1.040]; p<0.05 for regression coefficient difference between diabetes vs. no diabetes) (72). However, this study did not find significant associations for other pollutants such as PM_10_ and O_3_, perhaps due to estimating air pollution exposures using central monitors rather than patient residential addresses.

**Table 4:** Studies examining the increased CVD risk for people with diabetes exposed to air pollution.

First Author (Year)	Design	Location	Population/Health Data Source	Pollutants	Relevant Outcomes
Zanobetti & Schwartz (2000) (67)	Cross-sectional	IL, USA	Medicare claims data, years 1988-1994	24-hr PM_10_	2.01% greater hospital admissions for CVD in people with diabetes per 10 μg/m^3^ increase in PM_10_ vs. 0.94% greater CVD admissions for people without diabetes.
O’Neill et al. (2005) (68)	Cross-sectional	MA, USA	Boston-area residents, 270 adults	24-hr PM_2.5_, BC	7.6% decrease in nitroglycerin-mediated vascular reactivity and 10.7% decrease in flow-mediated reactivity in people with diabetes. Null findings for people without diabetes.
Park et al. (2005) (69)	Cross-sectional	MA, USA	VA Normative Aging study, 603 adults	4-24-48-hr PM_2.5_, PN, BC, O_3_, NO_2_, SO_2_, CO	Nonsignificant but marked trend toward reduced HRV in people with diabetes compared to people without diabetes.
Zeka et al. (2006) (70)	Case-crossover	United States	National Center for Health Statistics, 1,896,306 deaths	PM_10_ 0-3d before death	No significant effect modification of PM_10_-CVD death association by diabetes status.
Pope et al. (2006) (71)	Case-crossover	UT, USA	Intermountain Health Collaborative Study, 12,865 adults	PM_10_, PM_2.5_	Similar PM_2.5_ risk estimates for people with vs. without diabetes.
Peel et al. (2007) (72)	Case-crossover	GA, USA	Emergency department data, 4,407,535 visits	24-hr PM_10_, 8-hr O_3_, 1-hr NO_2_, SO_2_, CO	For increasing NO_2_ exposure, 15.6% greater odds of visit for dysrhythmia in people with diabetes compared to 1.4% greater odds in people without diabetes.
Pope et al. (2014) (73)	Prospective cohort	United States	American Cancer Society Cancer Prevention Study II, 669,049 adults with varying diabetes status at baseline	Monthly PM_2.5_	People with both diabetes and >75^th^ percentile for exposure to PM_2.5_ at highest risk of CVD death. No evidence of interaction between diabetes status and PM_2.5_ exposure.
Vora et al. (2014) (74)	Double-blind, randomized, crossover trial	NY, USA	19 adults with diabetes	PM_0.1_ vs. filtered air	8 beats/min greater heart rate in PM_0.1_ condition.

In addition to clinical outcomes, exposure to air pollutants has also been associated with subclinical effects. In 2005, a cross-sectional study of 270 adults in Boston found that, among subjects with diabetes, PM_2.5_ exposure was associated with a 7.6% decrease in nitroglycerin-mediated vascular reactivity [-12.8%, -2.1%], while black carbon exposure was associated with a 10.7% decrease in flow-mediated reactivity [-17.3, -3.5]. However, there was no such association among subjects without diabetes (68). That same year, a cross-sectional study of participants in the Veterans Administration Normative Aging Study observed that the association between PM_2.5_ exposure and reduced heart rate variability (HRV) was more pronounced in participants with diabetes. Indeed, the percent change in the standard deviation of normal-to-normal intervals (a measure of HRV) due to PM_2.5_ exposure, although not significant, was nearly 4-fold higher in participants with diabetes (-16.6% [-36.3, 9.2] compared to -4.7% [-11.4%, 2.6%]) (69). A double-blind, crossover exposure study of 17 never-smoker adults with diabetes found that 2 hours of controlled exposure to PM_0.1_ reduced heart rate variability (p = 0.014) and also increased average heart rate by approximately 8 beats per minute over a day after the exposure (74). These data point to the synergistic interaction between diabetes and air pollution in driving CVD.

Not all studies support an interaction between diabetes and air pollution. A case-crossover study examining emergency department visits for acute coronary syndrome in Utah found little difference in the PM_2.5_ risk estimate for people with diabetes compared to those without (71). A similar case-crossover study of death records from 20 cities in the United States found no significant effect modification of the PM_10_-CVD death association by diabetes status, although the point estimate for the association between PM_10_ and all-cause mortality was higher for people with diabetes compared to those without (70). Moreover, in an analysis of 22 years of follow up in the American Cancer Society Cancer Prevention Study II cohort, people with diabetes had a higher risk of CVD mortality at both high (HR 2.4 [2.3, 2.5]) and low PM_2.5_ exposure (2.2 [2.1, 2.3]) compared to people without diabetes. However, when comparing high to low PM_2.5_ exposure, the CVD mortality risk increase was similar in both groups, and formal tests of interaction between diabetes status and PM_2.5_ exposure were nonsignificant (73).

### 3.4 Environmental inequities contribute to unequal diabetes and cardiovascular disease risk

Consistently, research in the United States has shown that racial/ethnic minority communities, and individuals with low education and income, have greater diabetes prevalence (75) and mortality (76). Racial/ethnic minorities also develop diabetes at lower BMIs compared to white people, and the strength of the association between BMI and diabetes is weaker in racial/ethnic minorities, highlighting the complexity of factors that may contribute to this disproportionate burden (77). Recent work in the US and limited research in Asia and Africa has shown that low-SES communities are subject to higher air pollution exposures (78). In the United States, black and Hispanic minority groups are disproportionately exposed to the air pollution generated by the white majority (79). This disproportionate exposure translates into a greater burden of death due to air pollution. A retrospective cohort study of US Veterans Administration patients found that excess death due to PM_2.5_ exposure was disproportionately borne by black patients (55.2 deaths per 100,000 [50.5, 60.6]) compared to nonblack patients (51.0 [46.4, 56.1]), as well as by patients living in low SES counties (65.3 [56.2, 75.4]) compared to those living in high SES counties (46.1 [42.3, 50.4]). Notably, 99% of these excess deaths were due to PM_2.5_ concentrations below the US Environmental Protection Agency (EPA) recommended limit of 12μg/m^3^ (80). These findings are put into historical context when considering that historically redlined neighborhoods face greater PM_2.5_ and NO_2_ exposures compared to other communities in the same cities. Even within redlined neighborhoods, racial and ethnic disparities in air pollution exposure persist (81). Thus, multiple traditional and non-traditional risk factors are disproportionately concentrated in minority communities and may act in concert to further widen health disparities.

### 3.5 Inconsistencies in the data

As noted previously in this review, some studies have not detected an association between air pollution and incident or prevalent diabetes. Moreover, associations between long-term air pollution exposure and HbA1c were, though not entirely consistent, generally positive. Results for gaseous pollutants appear to be more heterogeneous than particulates; however, since there are correlations between these pollutants, it can be challenging to parse out outcomes for individual components in epidemiological studies. Some associations appear to be only in, or stronger in, subgroups; additionally, some effects appear to be attenuated by other factors such as medications (45). Regional, cultural, gender, socioeconomic or other differences in work or lifestyle can influence how people spend time in different geographical areas, thereby changing both pollution sources and exposures. Given the temporal and spatial heterogeneity of air pollution concentration and composition, accurate and precise exposures are extremely difficult to assess making some degree of variation in these results unsurprising. Lastly, although experimental studies suggested that having diabetes can exacerbate the cardiovascular derangements induced by air pollution exposure, and epidemiologic observations often reported greater associations between air pollution and death for people with diabetes, the evidence of an effect modification on air pollution-CVD death by diabetes status or an air pollution-diabetes interaction on CVD death remains lacking. The lack of detectable effect modification could be due to inaccurate reporting on surveys and death records. Alternatively, the majority of the effect of air pollution on CVD death may be attributable to air pollution promoting a cardiometabolic disease state. Thus, some of this effect could be lost when stratifying by diabetes status (73).

### 3.6 A word on the exposome

Although this review is concerned with the health effects of air pollution exposure, it is important to note that such exposure does not occur in a vacuum. Instead, air pollution exposure often co-occurs with a variety of other environmental exposures, such as noise pollution, nighttime light, and temperature, especially in urban areas (82). Originally conceived as a complement to the genome (83), the term “exposome” aims to capture the host of biological responses to the myriad environmental exposures experienced throughout the life course. In recent years, there has been a growing call to consider each environmental exposure in context of the entire exposome. Much of the prior observational analysis has focused on single exposures in isolation. To completely understand the health implications of environmental exposures, including air pollution, experts have argued in favor of advanced analytic methods that consider diverse, simultaneous exposures, their interactions, and their measurable biological effects (84). Such analytic methods will need to venture beyond standard univariate and multivariate regression models to tease out the likely non-linear effects of a multitude of co-occurring exposures (85). Taking a page from studies of genetic associations, a novel method known as environment-wide association study might identify the effects of mixtures of exposures (86). Future observational studies examining the air pollution, diabetes, and CVD link should take into account the exposome concept to more accurately reflect the reality of how people experience these exposures.

### 3.7 The global variability of air pollution and its health implications

It is worth noting that although air pollution is experienced by nearly all people globally, the burden of air pollution varies by region. Over the past two decades, average airborne PM_2.5_ concentrations have declined in North America, Europe, and East Asia, whereas the opposite has occurred in the Middle East, Africa, and South Asia (87, 88). Despite this increase in ambient PM concentration in the Middle East and North Africa, morbidity and mortality rates due to air pollution have decreased in these regions, which might be due to a declining rate of indoor fuel burning (89). Nevertheless, ambient air pollution remains an urgent public health concern, with approximately 22% of deaths due to ischemic heart disease and 21% of deaths due to diabetes attributed to air pollution in the Middle East and North Africa (89).

Among global regions, substantial differences have been noted in the magnitude of the association between higher PM exposure and increased mortality (90). Such differences are likely due to a variety of factors. Countries vary in the relative contributions of traffic, industry, and biomass burning to the generation of PM (91). These pollution sources differ in the exact chemical composition PM (92), which might explain the global differences in mortality risk due to PM exposure. Moreover, indoor burning of biomass fuel, such as wood, crops, and manure, for heating and cooking can drastically worsen indoor air quality (93). The greater indoor burning of such fuels in lower- and middle-income countries (LMIC) could alter the observed association between outdoor air pollution and mortality while also placing people in these countries at higher risk (90). As discussed previously in this review, LMIC face a greater projected increase in prediabetes and diabetes compared to high-income countries. Many of these countries also face trends of worsening air quality. Evidently, the combined epidemics of air pollution exposure and diabetes represent an urgent threat to global public health.

### 3.8 Conclusions from epidemiological data

Overall, the studies to date indicate that PM_2.5_, PM_10_, PN_AM_, black carbon, SO_2_, and CO may have deleterious effects on glucose homeostasis in the short- and long-term. Consistently, PM_2.5_ had the most consistently demonstrated effect on glucose across multiple populations. The inconsistent results for NO_2_ may be due to low diabetes event rate in the study subjects and/or low overall levels of NO_2_ leading to small effect size. Acute air pollution exposure is more strongly associated with increased fasting blood glucose, whereas chronic exposure has a stronger association with worsened HbA1c. Susceptible subgroups demonstrate stronger effects of air pollution on glucose metabolism as well as diabetes prevalence and incidence among those with overweight and obesity. Consequently, air pollution appears to have stronger effects on adverse CV outcomes among those with dysregulated glucose homeostasis.

While there is a growing body of work in this field, most epidemiological studies to date have been conducted in populations residing in the US, Canada, western Europe, and East Asia. Most of these regions have experienced improvements in air pollution levels in recent decades, while LMIC have experienced an increase in morbidity and mortality due to air pollution (94). With diabetes prevalence on the rise worldwide, more studies are needed to investigate the effects of worsening pollution on the metabolic health of people living in LMIC. Results from wealthier countries cannot be extrapolated to LMIC, given the known differences in air pollution exposures and population characteristics between these regions.

## 4 Shared mechanisms

While human epidemiological studies can identify associations, mechanistic evidence is important to define the affected biological pathways. Such data can assist in identifying susceptibility factors, specific pollutants to target with regulation, or molecular targets for pharmaceutical interventions. To date, mechanistic studies in this field include known exposure studies in cell lines and in animal models, exposure chamber studies, and natural experiments with humans. In this section, we will review these types of studies and summarize the identified mechanisms that underlie the associations between air pollution, dysregulated glucose metabolism, and increased CVD risk.

### 4.1 Mechanisms in animal studies

**Figure 2:**
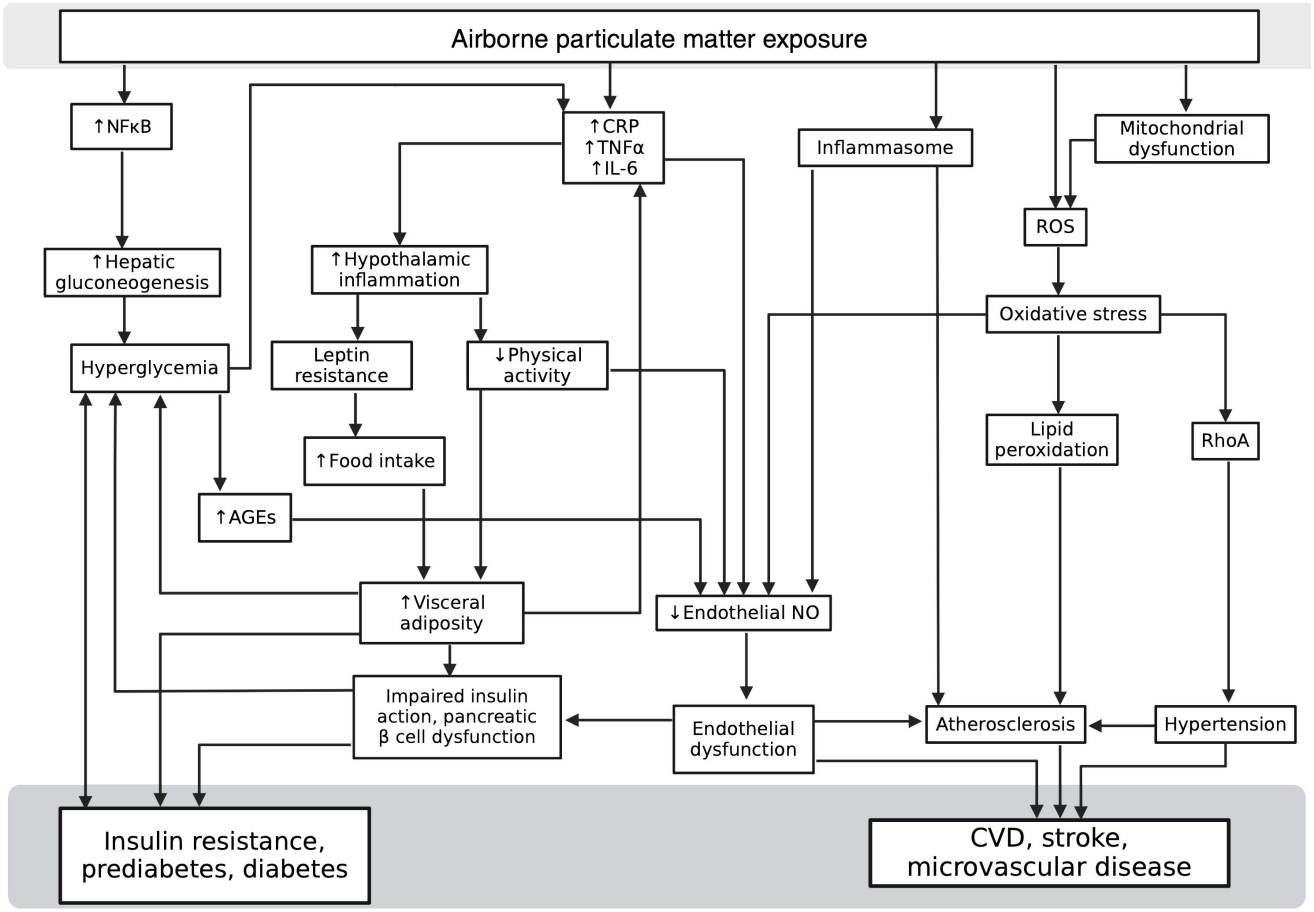
Multiple overlapping mechanisms by which air pollution, diabetes, and cardiovascular disease interact. (AGEs, advanced glycosylation end products; CRP, C-reactive Protein; CVD, cardiovascular disease; IL-6, Interleukin-6; NFκB, Nuclear factor kappa-light-chain-enhancer of activated B cells; NO, nitric oxide; RhoA, Ras homolog family member A; ROS, reactive oxygen species; TNFα, tumor necrosis factor alpha). Created with BioRender.com.

There are multiple convening pathways by which air pollution, diabetes, and cardiovascular disease interact (**Figure 2**). Much of the data to date suggests two primary culprits are inflammation and oxidative stress, themselves intertwined, which often form self-perpetuating feedback loops.

#### 4.1.1 Inflammation in *animal models*

##### 4.1.1.1 Hypothalamic inflammation, diabetes, and air pollution

Inflammation plays a dominant role in the development and progression of diabetes (95, 96). Hypothalamic inflammation is proposed to be a major driver of disorders of glucose homeostasis due to its role in regulating energy intake and expenditure via insulin and leptin (97). Animal models show that recurrent hypothalamic inflammation, via diet-induced increases in TNFα expression (98–100), leads to a dysregulated body weight set-point, driving increased energy intake and decreased energy expenditure. These behaviors serve to increase adiposity that further increases inflammation (101). These appear to be independent, yet synergistic, effects on inflammation. In fact, a study in a mouse model of diabetes and prediabetes suggested that differences in hypothalamic inflammation could be to blame for the observed variation in the onset and progression of prediabetes to diabetes within the group of mice (102).

The hypothalamus appears to be susceptible to the inflammatory effects of particulate matter pollution. A mouse model was exposed to PM_2.5_ or clean air, and inhibitor of nuclear factor kappa-B kinase subunit beta (IKK2), an NF-kB inhibitor that interferes with inflammatory signal transduction. Pollution-exposed mice treated with cerebral IKK2 demonstrated an attenuation of the insulin resistance shown in pollution-exposed mice not treated with IKK2; they also had evidence of inhibition of hepatic gluconeogenesis enzymes. Overall, this suggests that PM likely increases the production of these enzymes in part via inflammatory signaling through NF-kB (103). In a separate study, mice were fed a normal chow diet and exposed to PM_2.5_ or filtered air. After five days of PM_2.5_ exposure, there was chemical and histologic evidence of a heightened inflammatory response within the hypothalamus, with accompanying food-seeking, exercise-avoidant behavior changes, and adipose gain. After exposure to PM_2.5_ for twelve weeks, the mice developed increased toll-like receptor 4 (TLR4) and Ikbke (related to NF-kB) expression, leptin and insulin resistance, and a worsening of their energy homeostasis and development of frank obesity. In this study, knockdown of TLR4 and Ikbke completely attenuated the effects of PM_2.5_ exposure on leptin and insulin (104). Together, these suggest that hypothalamic inflammation could lie along a potential causal pathway between air pollution exposure and dysregulated glucose homeostasis.

##### 4.1.1.2 Inflammation, vascular disease, and air pollution

In addition to the hypothalamic inflammation, air pollution has been shown to exacerbate inflammation systemically. In airway epithelial cells and macrophages, O_3_ exposure has been shown to induce the production of inflammatory cytokines, interleukin-6 (IL-6), and interleukin-8 (IL-8) (105). Animal experiments show that excess glucose and triglycerides cause inflamed adipose tissue to secrete adipokines, driving insulin resistance and pancreatic β cell exhaustion, thereby exacerbating nutrient excess and leading to further inflammation (106). In a C57BL/6 mouse model, male mice fed a high-fat diet were randomly assigned to PM_2.5_ exposure or clean, filtered air. Compared to the control, clean air group, the mice in the exposed group developed elevated insulin resistance, increased visceral fat, and increased adipose inflammation (107). Furthermore, the exposed mice exhibited decreased vascular relaxation in response to insulin and acetylcholine, indicating insulin resistance (107). In mice, co-exposure to SO_2_, NO_2_, and PM_2.5_ increased circulating levels of the inflammatory molecules TNF-α, IL-6, and cyclooxygenase-2, while also dose-dependently increasing endothelin-1 and decreasing endothelial nitric oxide synthase, which reflect impaired endothelial function (108). This finding is consistent with prior animal research that implicates inflammatory cytokines in the impairment of vascular tone (109, 110).

Air pollution exposure may also potentiate atherosclerosis progression. PM_2.5_ exposure in mouse models accelerates atherosclerosis and increases inflammation compared to filtered air, with stronger effects in mice on a high-fat diet (111, 112). Notably, inflammasome activation plays a central role in this process (113). These results demonstrate the indirect effects of air pollution on vascular function. Interestingly, inhaled nanoparticles in rats accumulate in areas of vascular inflammation, including atherosclerotic plaques, suggesting the direct effects of PM exposure on vascular tissue may also be relevant. Together, these studies support that particulate matter pollution can accumulate in, and worsen the inflammation of, adipose and vascular tissue, potentially worsening already impaired vascular and endothelial function (114). Overall, animal experiments to date suggest that air pollution exposure may independently promote the development and worsening of both diabetes and atherosclerosis with a central role for inflammation in each of these processes.

#### 4.1.2 Oxidative *stress*

Separate from inflammation, air pollution exposure induces oxidative stress, which refers to the state of imbalance in reactive oxygen species (ROS) and antioxidant mechanisms such that ROS may induce damage to cellular structures or other biomolecules of importance (115). PM_2.5_ and PM_0.1_ were shown to accumulate in mitochondria, causing damage to the mitochondria and possibly potentiating the effect of ROS (37). Transition metals, present in PM_2.5_ and PM_0.1_, generate ROS at the particle surface, causing oxidative stress and mitochondrial damage (37). Furthermore, polycyclic aromatic hydrocarbons, quinones, and peroxyacetyl nitrate found in the organic carbon fraction of PM are potent inducers of oxidative stress (116, 117). Even O_3_, when dissolved in plasma, or serum, or saline, generates H_2_O_2_ (118).

Multiple studies *in vivo* and *in vitro* have shown that oxidative stress drives many of the vascular complications of diabetes (119). In particular, hyperglycemia appears to promote mitochondrial generation of superoxide (120), while interfering with this production attenuates the damaging effects of hyperglycemia on the endothelium (121). ROS also produce nitrotyrosine, which has been shown to accumulate in necrotic and apoptotic cardiac myocytes from patients with diabetes and from a rat model of diabetes (122). Generation of superoxide by NADPH oxidase also appears to play a significant role in the micro- and macro-vascular complications of diabetes (123).

Oxidative stress is extensively implicated in the development of CVD (124) and has been shown in animal models to play a key role in mediating the cardiovascular effects of air pollution exposure (125). After 10 weeks of exposure to 14.1 μg/m^3^ PM_2.5_ and PM_0.1_, rats had increased superoxide concentrations in their aortas with signs of oxidative stress due to both impaired endothelial nitric oxide synthase and impaired hepatic synthetic function. In these rats, ROS generation from both PM_2.5_ and PM_0.1_ activated RhoA, a known mediator of vasoconstriction and acute hypertension. RhoA activation correlated with an increased mean arterial pressure of the rats exposed to the PM versus control (126). Compared to clean air controls, rats exposed to concentrated PM for 5 hours had twice the amount of oxidative stress in cardiac tissue (127).

#### 4.1.3 Conclusions on mechanisms from animal studies

Numerous studies show increases in both inflammation and oxidative stress. The specific vascular impact of air pollution-related oxidative stress in animal models of diabetes has not been extensively studied. However, evaluating the evidence discussed in this section, it is reasonable to hypothesize that air pollution can exacerbate vascular complications of diabetes via increased oxidative stress. Air pollution-induced inflammation and diabetes may then synergistically exacerbate cardiac and vascular dysfunction, providing plausible causal explanations for the links between air pollution, diabetes, and cardiovascular disease in epidemiological studies.

### 4.2 Mechanisms in human studies

Human studies are limited in the ability to test exposures and outcomes ethically. Some experimental exposure studies in healthy volunteers have investigated the molecular mechanisms underpinning the adverse health effects associated with air pollution exposure. There have also been some epidemiological studies with molecular testing that is suggestive of mechanisms. While limited, these studies can confirm animal studies and validate these pathways in humans.

#### 4.2.1 Inflammation in *humans*

##### 4.2.1.1 Inflammation and diabetes

Inflammation appears to play a role in the pathogenesis of diabetes. Studies investigating this relationship have been outlined in **Table 5**. Multiple nested case-control studies have investigated the role of inflammatory cytokines in the development of diabetes. In the Women’s Health Study, there was an increased risk of developing diabetes for those in the highest vs. lowest quartile of baseline IL-6 (RR: 2.3 [0.9, 5.6]) and CRP (RR: 4.2 [1.5 – 12.0]) (128). Similarly, in the EPIC-Potsdam study, IL-6 and CRP were not only significantly correlated with HbA1c (0.099, p = 0.019, and 0.1, p = 0.017, respectively), but also with odds of developing diabetes (OR: 2.57 [1.24 – 5.47] and 1.9 [1.2 – 3.2], respectively) in models adjusting for traditional diabetes risk factors and HbA1c (129). A nested case-control analysis within a prospective study of 3,842 Swiss adults without baseline diabetes followed for 5.5 years on average showed that diabetes risk increased with highest vs. lowest quartile of baseline IL-6 (OR 1.58 [1.02 – 2.45]) and CRP (OR 4.63 [2.85 – 7.53]) (130). Finally, the Cardiovascular Health Study in the United States showed that having baseline CRP in the highest quartile was associated with increased odds of developing diabetes (OR 2.03 [1.44 - 2.86]) versus the lowest quartile (131). Related to metabolism and inflammation, the Framingham Heart Study showed a positive correlation between exposure to PM_2.5_ and SO_4_^2-^ with adipokines adiponectin and resistin, respectively.^1^

**Table 5:** Cited literature regarding the link between inflammation and diabetes in humans.

First Author (Year)	Design	Location	Population/Health Data Source	Biomarkers	Relevant Outcomes
Pradhan et al. (2001) (128)	Prospective, nested case-control	United States	Women’s Health Study, 188 cases, 362 controls	IL-6, CRP	Diabetes incidence RR 7.5 for IL-6, 15.7 for CRP
Spranger et al. (2003) (129)	Prospective, nested case-control	United States	EPIC-Potsdam, 192 cases, 384 controls	IL-1β, IL-6, TNF-α, CRP	Diabetes incidence OR 2.57 for IL-6, 1.9 for CRP. No association for TNF-α, IL-1β
Marques-Vidal et al. (2012) (130)	Prospective	Switzerland	CoLaus Study, 3,842 adults, no baseline DM	IL-1β, IL-6, TNF-α, CRP	Diabetes incidence, unadjusted OR 1.58 for IL-6, 4.63 for CRP. No significant associations for any biomarker after adjustment.
Barzilay et al. (2001) (131)	Prospective	United States	Cardiovascular Health Study, 4,481 older adults	CRP, WBC, platelets, fibrinogen, factor VIIIc, albumin	Diabetes incidence OR 2.03 for CRP, no associations for other biomarkers
Li et al. (2018) (50)	Prospective cohort	United States	Framingham cohorts, 5,958 adults, no baseline DM	adiponectin, resistin, leptin	Positive association between 7-day PM_2.5_ and adiponectin, 7-day SO_4_^2-^ and resistin; negative association between 7-day NO_x_ and adiponectin
Krämer et al. (2010) (41)	Prospective cohort	Germany	SALIA cohort, 1,775 adult women, no baseline DM	C3c	C3c significantly associated with PM_10_ and diabetes incidence, HR 1.12 per 10 mg/dL increase

Outcomes column reports results for fully adjusted models when applicable. Means reported for significant results.

The hypothalamic inflammation linked with obesity and diabetes in mouse models recapitulates in humans with multiple magnetic resonance imaging (MRI) studies (100, 132–135). However, while growing evidence implicates hypothalamic inflammation in abnormal glucose homeostasis in humans, we are aware of no human studies investigating this as a direct result of air pollution exposure. Such investigation, using quantitative MRI methods similar to the other studies in this section, may confirm the air pollution and hypothalamic inflammation link observed in animal studies.

##### 4.2.1.2 Inflammation, atherosclerosis, and air pollution

There is a well-known association between inflammation and clinical atherosclerosis (136, 137). Multiple recent reviews (138–142), as well as a meta-analysis (143), discuss the associations between inflammatory biomarkers and air pollution exposure. The most commonly studied inflammatory biomarkers include CRP, IL-6, and TNF-α, but others demonstrate associations with pollution exposure. Literature exploring air pollution promoting CVD via inflammation has been listed in **Table 6**. Experimental exposure studies in healthy volunteers have shown increases in biomarkers of inflammation with controlled exposure to urban air pollution (147, 148), but not wood smoke (149), indicating the importance of pollution composition. Gene expression studies show increased activation of anti-inflammatory pathways that support inflammation as a mediator for air pollution exposure-related adverse effects (150). A study of traffic-related air pollution exposure in adolescents and young adults with type 1 diabetes showed increases in IL-6 and CRP as well (151).

**Table 6:** Cited studies investigating the impact of air pollution driving CVD via inflammation.

First Author (Year)	Design	Location	Population/Health Data Source	Pollutants	Relevant Outcomes
Azzouz et al. (2022) (144)	Cross-sectional	Sweden	Malmö Diet and Cancer, Cardiovascular Subcohort, 6,103 adults	Annual PM_2.5_, PM_10_, NO_x_	PM_2.5_ and PM_10_ associated with increased ceruloplasmin, alpha-1-antitrypsin. PM_2.5_ associated with Lp-PLA_2_, NLR, C3, haptoglobin, orosomucoid. No associations for NO_x_
Abohashem et al. (2021) (145)	Retrospective cohort	United States	Massachusetts General Hospital, 503 adults	Annual 24-hr PM_2.5_	PM_2.5_ associated with bone marrow and splenic activity, arterial inflammation, and MACE (HR 1.404)
Brook et al. (2008) (48)	Randomized, double-blind, crossover	Canada	31 healthy adults	2-hr exposure to PM_2.5_, O_3_	PM_2.5_ and O_3_ increased WBC, decreased FMD. PM_2.5_ increased neutrophils and diastolic BP.
Zhang et al. (2023) (146)	Panel	China	45 healthy college students	1-, 2-, 3-day PM_2.5_ and metal fractions	Association between metal fractions and sCD36, CRP, and pulse pressure.

Outcomes column reports results for fully adjusted models when applicable. Means reported for significant results.

Several recent studies have investigated the role of inflammation as a mediator of air pollution-linked CVD risk. Inflammation and CVD biomarkers were examined in a cross-sectional analysis of 6,103 participants with and without CVD in a Swiss cohort. A 5 μg/m^3^ increase in annual mean PM_2.5_ exposure was associated with increased ceruloplasmin (β = 0.1328 [0.0898, 0.1757]) alpha-1-antitrypsin (β = 0.105 [0.0564, 0.1537]), Lp-PLA_2_ (β = 0.085 [0.0303, 0.1397]), neutrophil-leukocyte ratio (0.074 [0.0054, 0.14]), C3 (β = 0.1618 [0.1302, 0.2071]), haptoglobin (β = 0.0981 [0.0075, 0.1886]), and orosomucoid (β = 0.205 [0.1505, 0.2595]) (144).

Increased exposure to metals from measured PM_2.5_ is associated with increased inflammatory biomarker sCD36, which in turn has a significant mediating effect on the association of these metals with pulse pressure, providing further evidence that inflammation appears to occur upstream of CVD in the context of PM_2.5_ exposure. No significant associations were found for total PM_2.5_, highlighting that the individual components of PM_2.5_ likely have differential effects on inflammation and oxidative stress in humans, which should prompt further investigation (146).

Vascular inflammation is seen in subclinical atherosclerosis using PET/MRI hybrid imaging techniques (152), implying that inflammation has a role early in the development of the disease. Inhalable particles likely have direct local effects on arterial disease and indirect systemic effects. For example, when human volunteers at risk for stroke were exposed to inhalable gold nanoparticles, these particles were then detected in their diseased carotid arteries (153). Advanced imaging techniques have been implemented to investigate a direct link between PM, inflammation, arterial damage, and CVD outcomes in humans (145), supporting likely direct vascular inflammatory effects of air pollution exposure. These studies suggest air pollutants may act via both systemic and direct vascular inflammatory actions to promote CVD, corroborating the epidemiological studies and animal models. Additional research may more fully elucidate which specific components of pollutants may act on which pathways to identify potential targets for intervention.

#### 4.2.2 Oxidative *stress* in *humans*

The effect of air pollution on oxidative stress, demonstrated robustly in animal models, has had inconsistent evidence in humans, likely because measuring oxidized DNA and lipids in humans can be a technological challenge (154, 155). However, a meta-analysis of studies examining oxidized DNA and lipids in subjects exposed to air pollution that had minimal measurement error demonstrated a consistent association between PM_2.5_ and these measures of oxidative stress (156).

In particular, a study in healthy adults and adults with diabetes found that inducing labile blood glucose via clamp resulted in elevations of the markers of oxidative stress plasma 3-nitrotyrosine and PGF2α, a decrease in NO synthesis, as well as impaired endothelial function in the presence of vasodilating agents (157). Furthermore, peroxynitrite, generated by the reaction of superoxide and endothelial NO, has been detected at elevated concentrations (158) and shown to induce platelet damage in the blood of patients with diabetes (159).

#### 4.2.3 Inflammation and oxidative stress promote endothelial dysfunction

Endothelial dysfunction mainly refers to the impairment of endothelium-mediated relaxation of vascular tone. Human studies have consistently reported an association between traditional cardiovascular risk factors and endothelial dysfunction (160). Furthermore, endothelial dysfunction can predict progression and long-term outcomes of coronary heart disease (161).

Inflammation is known to promote endothelial dysfunction in humans (110, 162) in a variety of disease states, including obesity and diabetes (163). This link between inflammation and endothelial dysfunction appears to be present in the context of air pollution exposure. In a study of healthy volunteers with experimental exposure to PM_2.5_, higher TNF-α just after air pollution exposure was associated with poorer endothelial function a day later, suggesting that endothelial dysfunction seen with air pollution may be mediated by an inflammatory cascade that begins acutely during and after exposure, even in people that are free of diagnosed disease (164). Research in animals has demonstrated that the production of ROS by inflammatory cytokines, as well as the formation of advanced glycation end products (AGEs), alter the availability of endothelial NO, leading to endothelial dysfunction (110). ROS occurs in hypertension, hyperlipidemia, and diabetes, providing a likely explanation for the link between these traditional risk factors and endothelial dysfunction (165). The mechanistic inclusion of AGEs in this pathway is of special interest, as diabetic hyperglycemia promotes the endogenous production of AGE by irreversibly glycating tissue proteins and lipids (166).

Recently, endothelial dysfunction of arterioles and microvessels was demonstrated to predict the development and progression of diabetes in a German prospective cohort study of 15,000 adults without baseline diabetes or prediabetes (167). Mechanistically, microvascular endothelial dysfunction may impair insulin action in skeletal muscle and favor blood flow to nonnutritive tissues, thereby promoting hyperglycemia (168). Moreover, experiments in animal models have demonstrated that microvascular disease in pancreatic ß cells may drive the pathogenesis of diabetes (169, 170).

Lastly, accumulating evidence has demonstrated a link between air pollution and endothelial dysfunction mediated by inflammation and oxidative stress (171). Taken together, it appears that endothelial dysfunction may partly explain the air pollution-diabetes link while also providing a mechanism for the accelerated development of CVD in people with diabetes exposed to air pollution.

### 4.3 Conclusions from mechanistic data

Systemic inflammation and oxidative stress, converging at endothelial dysfunction, represent common mechanisms whereby air pollution induces glucose dysregulation and exacerbates CVD risk. Due to its role in the onset and progression of both diseases, air pollution represents a critical target in promoting the health of the public and individuals. The next section will explore interventions directed toward mitigating the effects of inflammation and oxidative stress.

## 5 An eye toward prevention

Despite inconsistencies in the observational literature, the overall balance of evidence across all the tiers of evidence quality supports a deleterious effect of short- and long-term air pollution exposure on metabolic health. Given that the global population will remain exposed to air pollution, for which a completely benign dose has yet to be established, efforts have been made to counter these adverse effects. This section will detail the research concerning such interventions, with close attention paid to barriers to effective implementation.

### 5.1 Interventions to reduce air pollution as driver of CVD risk in people with diabetes

There is a burgeoning body of literature demonstrating that the use of portable air cleaners (PAC), which are known to reduce PM_2.5_ exposure, can reduce serum concentrations of CRP, IL-6, and TNFα. An overview of this research can be found in a recent systematic review and meta-analysis written by our group (172). PACs may also reduce blood pressure (173, 174). Given the importance of inflammation in CVD progression in diabetes, interventions to attenuate the upregulation of these pathways are appealing. However, most of the trials that intervened with PACs were conducted in healthy volunteers for short periods of time and in very controlled settings. Functioning similarly to PACs, a series of trials that used N95 respirators on participants in China demonstrated benefits on systolic blood pressure, HRV, and IL-1 (175).

The testing of interventions to ameliorate the adverse effects of air pollution exposure on glucose metabolism has been limited. A randomized, double-blind crossover trial in 55 healthy college students residing in Shanghai placed sham or real air purifiers in participants’ dormitories for 1 week, followed by a 17-day washout period, then 1 week of the alternate treatment. The investigators found that serum glucose, glucose-6-phosphate, insulin, and HOMA-IR were lower during the real air purification period compared to the sham period (59).

Diet patterns and nutritional supplementation with antioxidants or vitamins have been examined for their potential to protect against the adverse cardiometabolic effects of air pollution. Numerous studies have investigated the anti-inflammatory effects of dark chocolate (176–181). Supplementation with L-arginine has been shown to mitigate air pollution-related blood pressure increases among adults with hypertension (182), while in adults with diabetes, L-arginine was shown to improve glucose control, blood pressure, and forearm blood flow (183). Vitamin E has also been studied and shown *in vitro* to reduce inflammatory biomarker expression after PM2.5 exposure to endothelial cells (184) and to reduce oxidative stress in humans with occupational exposures to air pollutants (185, 186). In a cross-sectional study of 47,000 adults, those in the highest quartile of compliance to the Dietary approaches to stop hypertension (DASH) diet had no significantly increased risk of PM_2.5_-associated hypertension. In contrast, the lowest quartile had significantly increased risk (OR: 1.20 [1.10, 1.30]) (187). While generally low-risk, none of these studies were adequate to conclusively recommend specific dietary interventions for protection against the adverse cardiometabolic effects of air pollution. However, they are suggestive of potential options that warrant further investigation.

### 5.2 Other interventions to reduce the cardiovascular harms of air pollution

Although the literature investigating interventions to reduce the cardiovascular harms of air pollution exposure in persons with diabetes may be limited, there are additional interventions that are low-risk and readily accessible. These other interventions may provide protection against air pollution exposure or mitigate its harms, despite a weaker evidence base compared to PACs.

First, observational evidence suggests that central air conditioning might mitigate the adverse cardiovascular effects of PM exposure (188–191), even though the filters commonly used in air conditioning systems are less efficient at removing airborne particulate matter compared to HEPA filters. Thus, people with prediabetes or diabetes could be encouraged to use central air conditioning, if accessible and affordable, instead of electric fans and especially instead of opening windows for indoor temperature regulation. The results of a few experimental studies also support the use of in-vehicle air conditioning to reduce air pollution exposure while driving (192–194).

Second, the use of cigarettes and other combustible tobacco products indoors generates smoke that reduces indoor air quality (195, 196). Residue from cigarette smoke can adhere to indoor surfaces, creating thirdhand smoke that may continue to harm health after a smoking session has ended (197). Furthermore, although the health effects of electronic cigarettes are still under active investigation, electronic cigarette vapors contain some of the same pollutants as tobacco smoke (198) and therefore may also worsen air quality when used indoors. Tobacco product and electronic cigarette cessation should be strongly encouraged in all people, especially those with diabetes. However, if a person with diabetes is unable to quit, they should be counseled to avoid smoking indoors.

Other practical advice includes limiting outdoor activities during periods of poor air quality. The Air Quality Index, an easily understandable scale that describes outdoor air quality (199), is available on the internet for many cities around the world, especially in North America, East Asia, and Europe. People with diabetes can be advised to regularly check the air quality index (AQI) for their location and adjust activity accordingly. Keeping windows closed can also mitigate exposure to poor outdoor air quality, as can the avoidance of walking beside roads with heavy traffic.

### 5.3 Policy implications and public health initiatives for prevention

Given the worldwide contributions of traffic, industry, and biomass burning to the generation of ambient PM, policies that address these sources would reduce the ambient air pollution in urban environments (91). Furthermore, policies aimed at improving capture of industrial air pollution, developing more efficient industrial and agricultural systems, promoting electrification of motor vehicles, decreasing meat consumption, and reducing carbon emissions have been identified as feasible ways to improve global air quality within the next few decades if sufficient political will is generated (200).

Regular use of screening tests such as HbA1c and fasting plasma glucose alone do not appear sufficient to identify all people at risk for diabetes and its complications (201), therefore, diabetes prevention would likely benefit from population-level interventions. Cross-sectional evidence suggests that policies and public health initiatives that aim to improve the walkability of urban spaces and access to green space should be pursued (202). Although enhancing access to healthy food might theoretically reduce the population risk for diabetes (203), the evidence supporting such an initiative is limited due to relatively few studies and heterogenous measures of the food environment (202, 204).

### 5.4 Prevention conclusions

Overall, there is a dearth of data on individual-level interventions to prevent PM-related CVD in people with diabetes. PACs have the most robust experimental evidence to support their use to lower blood pressure, reduce inflammation, and potentially improve glucose control. However, whether PACs can reduce the macrovascular or microvascular complications of diabetes is unknown. Efforts are ongoing to regulate pollutant concentrations on a societal level, but more research is needed to identify susceptible subgroups and effective interventions for them. Avoiding traffic exposure, closing windows, and using air conditioning at home and in vehicles are commonsense actions unsuited for a clinical trial. Thus, data on these preventive strategies are limited (205). However, that should not preclude recommending these low-risk interventions, particularly for those at increased risk.

## 6 Summary of key points

- Air pollution exposure, especially fine particulate matter, is known to increase the risk of incident CVD and worsened CVD outcomes. There is no known safe dose of air pollution.
- Air pollution exposure increases the risk of incident diabetes and prediabetes in diverse populations and perturbs glucose homeostasis.*
- Prediabetes and diabetes confer an increased susceptibility to the cardiovascular harms of air pollution exposure.
- Air pollution exposure promotes local and systemic inflammation, which exacerbates atherosclerosis progression as well as endothelial dysfunction. In animal experiments, air pollution contributes to ROS formation and excess oxidative stress, as well as hypothalamic inflammation which may promote excess nutrient intake and resultant diabetes.
- Inflammation and oxidative stress are associated with dysregulated glucose metabolism in humans and animals. Hyperglycemia promotes further oxidative stress and inflammation, which may explain the progression from prediabetes to diabetes as well as the well-known increased CVD risk observed in people with diabetes.
* Mechanistic evidence supports the role of inflammation, oxidative stress, and hyperglycemia in the development of endothelial dysfunction. Air pollution and diabetes are both associated with endothelial dysfunction, which has been shown to predict CVD outcomes and incident diabetes.
- The study of interventions in people with diabetes to reduce the CVD risk due to air pollution has been limited. Some evidence points to the potential usefulness of portable air cleaners. Suggestive evidence supports further research into the effects of certain dietary and nutritional supplement interventions.

## 7 Conclusion

The importance of minimizing the impact of air pollution on a global scale cannot be overstated. The impact of air pollution on driving both the development of diabetes and exacerbating CVD risk in patients with diabetes is a topic that needs more research to reach a complete understanding of the interactions and mechanisms at play. Although there is heightened awareness of the adverse health effects of air pollution, further study on preventive strategies in people across the spectrum of dysregulated glucose homeostasis is greatly needed. An improved understanding of the mechanisms by which air pollution, diabetes, and cardiovascular disease interact would hasten the development of interventions to minimize the risks of exposure and slow disease progression. Furthermore, insights from this would greatly benefit a range of parties, including individuals concerned about their risks, healthcare providers wanting to provide optimal care and recommendations, and governments aiming to promote public health.

## Author contributions

LJB: Writing – original draft, Writing – review & editing, Visualization. SW: Writing – original draft, Writing – review & editing, Conceptualization, Visualization, Supervision. CL: Visualization, Writing – original draft. JA: Conceptualization, Writing – review & editing. JN: Conceptualization, Supervision, Writing – review & editing.

## Glossary

**Table:**

AQI	air quality index
BC	black carbon
BP	blood pressure
C3c	complement component 3
CVD	cardiovascular disease
DM	diabetes mellitus type 2
FBG	fasting blood glucose
FMD	flow-mediated dilation
GDM	gestational diabetes mellitus
HOMA-IR	homeostasis model assessment of insulin resistance
HRV	heart rate variability
IL-1β	interleukin-1 beta
IL-6	interleukin-6
IL-8	interleukin-8
LMIC	lower- and middle-income countries
LOS	length of stay
Lp-PLA2	lipoprotein-associated phospholipase A2
MACE	major adverse cardiovascular events
NFκB	Nuclear factor kappa-light-chain-enhancer of activated B cells
NLR	neutrophil-lymphocyte ratio
OM	organic matter
PAC	portable air cleaners
PM	particulate matter
PM_2.5_	fine particulate matter
PM_10_	coarse particulate matter
PM_2.5-10_	PM between 2.5 and 10 μm in aerodynamic diameter
PN_AM_	PM between 0.1-1 μm in aerodynamic diameter
ROS	reactive oxygen species
sCD36	soluble CD36
SES	socioeconomic status
TLR4	toll-like receptor 4
WBC	white blood cell count
WHO	World Health Organization
